# The Role of Diet on Insulin Sensitivity

**DOI:** 10.3390/nu12103042

**Published:** 2020-10-04

**Authors:** Maria Mirabelli, Diego Russo, Antonio Brunetti

**Affiliations:** Department of Health Sciences, University “Magna Græcia” of Catanzaro, Viale Europa, 88100 Catanzaro, Italy; maria.mirabelli@unicz.it (M.M.); d.russo@unicz.it (D.R.)

Growing evidence shows that dietary composition has a marked impact on the risk of developing obesity, type 2 diabetes (T2D), cardiovascular disease (CVD), certain types of endocrine cancer and many other intertwined metabolic and reproductive disorders, all featured by insulin resistance (IR) [1]. IR is an increasingly common pathological condition in Westernized populations [2], as well as in ethnic minorities [3], resulting from an attenuated response to insulin action in peripheral target organs and tissues, which include mainly, but not exclusively, skeletal muscle, fat and liver. Even though many molecular processes causing or modulating insulin sensitivity have been identified over the past few decades, there is neither consensus on the etiology of IR in obese individuals [4], nor a clear elucidation of its mechanistic connections with different eating habits and food components. The purpose of this Special Issue is to collect original clinical and preclinical research, as well as comprehensive reviews of the literature, outlining the dietary and nutraceutical modulation of insulin sensitivity and their implications in metabolic functions. New information has been added in this field by means of ten articles, with eight original articles and two extensive narrative reviews, summarized herein.

In recent decades, researchers have emphasized a connection between increased ceramides abundance in muscle, liver and adipose tissue, and impaired response to insulin. Coincidently, the accumulation of ceramides in key metabolic tissues, mostly due to excessive dietary fat and calorie intake, has been recognized as a potential target for novel preventive and/or therapeutic strategies in obesity and related disorders [5]. Ceramides are central molecules of sphingolipid metabolism, which can act at different cellular compartments (e.g., plasma membrane, endoplasmic reticulum, and mitochondria), with detrimental effects on insulin signaling and glucose homeostasis [5]. Teng and colleagues demonstrated that sulforaphane, a naturally occurring isothiocyanate compound in cruciferous vegetables such as broccoli and cabbage, can dose-dependently recover insulin signaling in in vitro models of hepatic IR by exerting inhibitory activities on the expression of *SPTLC3*, the gene encoding the third subunit of the rate-limiting enzyme of ceramide biosynthesis (serine palmytoiltransferase, SPT) [6]. These results were replicated in a high-fat-fed murine model of IR and T2D, in which sulforaphane treatment improved glucose tolerance and insulin sensitivity, attenuated hepatic ceramide biosynthesis and reduced hepatic triglyceride levels, thus suggesting in vivo protection from hepatic steatosis [6]. Likewise, Lepore and colleagues detailed the protective actions of oleacein, one of the prominent phenol constituents of extra virgin olive oil (EVOO), in alleviating the metabolic alterations occurring under in vivo chronic high-fat feeding conditions [7]. Daily oral oleacein treatment reduced adiposity and prevented the occurrence of steatohepatitis in a high-fat-fed mouse strain genetically prone to develop obesity [8]. Additionally, in animals treated with oleacein, the abdominal adipose tissue was characterized by a less pronounced inflammatory infiltrate, low expression levels of fibrosis marker genes and reduced adipocyte cell size [7]. This study proved that oleacein possesses the ability to regulate key adipose genes involved in adipogenesis and lipid accumulation in a targeted and highly sophisticated way, resulting in distinct biological implications for the developmental and maintenance stages of obesity [7]. On a separate note, Cassano and colleagues evidenced that the glycemic derangement typical of T2D, which was induced by chronic high-fat feeding and streptozotocin injections in Wistar rats, could be ameliorated by treatment with ranolazine, a relatively new anti-arrhythmic and anti-anginal compound [9]. Furthermore, ranolazine showed promising potentials for neuroprotection in obesity and T2D, and improved the body composition of high-fat-fed animals by effectively increasing their lean body mass [9]. In this context, increasing evidence is linking adiposity to the impaired brain structure and decreased insulin sensitivity of this target organ in patients with excessive body weight. Additionally, obese individuals suffering either from T2D or milder forms of hyperglycemia are exceedingly prone to cognitive decline and dementia, showing progressive deficits over time [1]. Although this was not assessed with ex vivo histological analyses, results from in vivo behavioral and cognitive testing procedures suggest that ranolazine might effectively counteract the metabolic and structural brain disturbances induced by a high-fat diet and T2D [10]. 

Gender-based benefits of intensive lifestyle changes in preventing T2D and the adverse pregnancy outcomes of gestational diabetes (GDM) were an important aspect of this Special Issue. The milestone Diabetes Prevention Program (DPP) demonstrated that in a multi-racial population, engaged in a nutritional, physical and behavioral program aimed at losing at least 7% of body weight with extensive centralized training and support, the 3-year incidence of T2D was more than halved [11]. However, cultural differences and traditions, such as those typical of the Arab countries, can profoundly affect the feasibility of a DPP-based educational model, and appropriate modifications to the former intervention should be considered in this setting. By involving primary health facilities, Al-Hamdan and colleagues provided evidence of the efficacy and sustainability of a 6-month personalized lifestyle intervention in at-risk Saudi women with prediabetes [12]. Because of gender segregation, obligations concerning dress code (e.g., wearing abaya or full-body garments) and restrictions in outdoor activities, women in Saudi Arabia are particularly predisposed to sedentary lifestyle and T2D development [13]. One-on-one dietary counseling and an on-demand support system delivered by primary care personnel, even if coupled with limited adherence to physical activity and only modest weight loss after 6 months of lifestyle intervention, were more effective than standard care in improving glycaemic and cardiometabolic profiles in this target group [12]. The beneficial effects of an intensive lifestyle intervention, starting in early pregnancy and made of a calorie-restricted, low-glycemic-index, low-saturated-fat diet combined with moderate intensity physical activity (30 min of walking at least four days a week) were evaluated by Menichini and colleagues [14]. In obese singleton pregnant women with IR, this early intensive lifestyle intervention did not reduce the incidence rate of GDM with respect to standard care; however, mean glucose values at 75 g oral glucose tolerance test (OGTT), performed at 16–18 and/or 24–28 weeks of gestation in agreement with Italian guidelines, were significantly lower, as well as the birth rate of large-for-gestational-age (LGA) babies [14]. There is evidence that an excessive shunting of maternal nutrients across the placenta accelerates fetal growth and adipose tissue accretion from the early stages of pregnancy, and this increases the risk of macrosomia [15]. At the same time, fetal growth restriction due to maternal undernutrition during pregnancy may end up in small-for-gestational-age (SGA) births, negatively affecting the perinatal morbidity and mortality, as well as the longer-term health, of newborns [16]. Still, in obese women with IR, repeatedly identified at higher risk for GDM complications when compared to their normal weight counterparts [15], the early lifestyle intervention with moderate reduction in caloric intake presented by Menichini and colleagues did not increase the risk of giving birth to SGA babies [14]. A different shade of evidence for the insulin-sensitizing effects of a low glycemic index diet was added by the work of Gao and colleagues [17]. Compared to a typical hospital diet, a low glycemic index diet that emphasized consumption of pulses improved both the indicators of insulin sensitivity/resistance (e.g., Matsuda index, homeostatic model assessment of insulin resistance, and HOMA-IR) and the biochemical markers of bone reabsorption (e.g., amino-terminal crosslinked telopeptides of type 1 collagen, NTx) in healthy participants subjected to four days of bed rest [17]. When forced to undergo prolonged bed rest due to traumatic injuries, major surgical procedures or a number of diverse medical and neurological reasons, patients experience substantial muscle wasting and a temporary exacerbation of IR, which significantly worsens the impairments in lipid and glucose metabolism [18]. Given that bone and glucose metabolism are closely related to each other, and that a preserved insulin signaling exerts a positive effect on bone strength and turnover [19], nutritional strategies, capable of mitigating a bed-induced IR state, should be carefully considered within the hospital setting as a means for improving patient outcomes and risks of bone loss. Quite the opposite, the crossover trial of Nichol and colleagues investigated the impact of an acute exposure to sucralose, one of the most popular and controversial calorie-free artificial sweeteners commercially available, on postprandial metabolic responses [20]. Indeed, while there is a general consensus that overconsumption of sugar-sweetened foods and drinks contributes to the high global prevalence of obesity and T2D, the literature contains conflicting results about whether or not a similar causal relationship would exist for artificial sugar-substitutes. Very recently, it has been reported that prolonged exposure to sucralose-sweetened drinks containing maltodextrin (a carbohydrate) decreases insulin sensitivity and alters brain and sensory pathways of sweet taste in healthy participants [21]. In this regard, as evidenced in this Special Issue, the acute ingestion of sucralose before an OGTT could differentially affect glucose and insulin responses in individuals with normal body weight with respect to those with obesity, probably due to variations in intestinal permeability. Intriguingly, even perception of sucralose taste impaired insulin responses to OGTT, underscoring the potential of artificial sweeteners to cause profound harm to metabolic health [20]. Finally, Weiller and colleagues detailed the effects of butaphosphan, an organic source of phosphorus commonly used in veterinary medicine, on insulin sensitivity and glucose metabolism in mice on acute food restriction while receiving either a normal- or a high-calorie diet [22]. Approved for use in dairy cows during the postpartum period of negative energy balance in order to avoid an excessive mobilization of body fat, butaphosphan exerts a variety of metabolic actions that depend, at least in part, on the type of animal feeding. Overall, this study demonstrated that butaphosphan treatment could preserve adipose tissue mass and increase blood glucose levels in food-restricted animals by promoting an IR state [22], which is consistent with human and animal evidence for modulatory roles of dietary phosphate and serum phosphorus on insulin signaling [23].

With regard to the narrative reviews included in this Special Issue, the one from Wali and colleagues critically addressed the cardiometabolic effects associated with high-fat dietary regimens and the underlying fundamental mechanisms [24]. Although not univocal, most epidemiological evidence links the intake of dietary saturated fats with T2D and CVD, which are generally accepted as major complications of IR. As extensively detailed, the subcellular abundance of diacilglycerol (DAG) and/or ceramides, both causing a direct loss of insulin signaling in mechanistic studies, inversely correlates with insulin sensitivity in target organs [24]. Moreover, the differential impact of specific acyl chain length ceramides on insulin homeostasis and functions represents an emerging concept in the nutritional regulation of glucose metabolism [5]. However, not all dietary fats have detrimental effects on insulin sensitivity. When compared to a low-fat diet, adherence to a Mediterranean-based regimen, particularly rich in unsaturated fats from EVOO or nuts, was associated with a significant reduction of CVD events and related mortality (approximately 30%) [25]. Other than basic nutrition and unsaturated fatty acids, the Mediterranean Diet provides several plant-based functional foods and nutrients that have been shown to have beneficial effects on insulin sensitivity and metabolic health, reversing IR states and certain hard-curative disease traits in patients with obesity, T2D, CVD, steatohepatitis, cognitive decline, endocrine-related cancers and polycystic ovary syndrome (PCOS) [1]. We had the opportunity to gather epidemiological and mechanistic evidence about the role of the Mediterranean Diet and selected nutritional supplements on the abovementioned IR-related diseases in a detailed review. Unsaturated fatty acids, flavonoids, anthocyanins and other polyphenols were inevitably in the spotlight [1].

Either individually or collectively, the studies included in this Special Issue produce new insights into the role of diet on insulin sensitivity and provide results of considerable value for health-policy-making processes. Although far more research is needed to confirm the findings of these works, this background knowledge will open new avenues for investigation and, possibly, affect clinical nutrition practice within a short time.
